# DNA Methylation Readers and Cancer: Mechanistic and Therapeutic Applications

**DOI:** 10.3389/fonc.2019.00489

**Published:** 2019-06-11

**Authors:** Niaz Mahmood, Shafaat A. Rabbani

**Affiliations:** Department of Medicine, McGill University Health Centre, Montréal, QC, Canada

**Keywords:** methyl-binding proteins, cancer, epigenetics, DNA methylation, MBD

## Abstract

DNA methylation is a major epigenetic process that regulates chromatin structure which causes transcriptional activation or repression of genes in a context-dependent manner. In general, DNA methylation takes place when methyl groups are added to the appropriate bases on the genome by the action of “writer” molecules known as DNA methyltransferases. How these methylation marks are read and interpreted into different functionalities represents one of the main mechanisms through which the genes are switched “ON” or “OFF” and typically involves different types of “reader” proteins that can recognize and bind to the methylated regions. A tightly balanced regulation exists between the “writers” and “readers” in order to mediate normal cellular functions. However, alterations in normal methylation pattern is a typical hallmark of cancer which alters the way methylation marks are written, read and interpreted in different disease states. This unique characteristic of DNA methylation “readers” has identified them as attractive therapeutic targets. In this review, we describe the current state of knowledge on the different classes of DNA methylation “readers” identified thus far along with their normal biological functions, describe how they are dysregulated in cancer, and discuss the various anti-cancer therapies that are currently being developed and evaluated for targeting these proteins.

## Dna Methylation And Cancer: An Introduction

The identity, behavior, and functionality of cells are dictated by the set of genes that are expressed at a given time. These genes are under tight regulation by different regulatory factors which makes sure that cells behave and function normally. However, in cancer, the cells lose their ability to behave normally which can be seen and measured by the qualitative and quantitative changes in the expression pattern of the active genes (1, 2). Historically, much attention has been given to the qualitative changes of gene expression which mainly focuses on the gene sequence-based alteration in the tumor cells (1). Therefore, cancer has been classically known as a predominantly genetic disease (3). However, the realization that quantitative changes in gene expression, due to different types of epigenetic modification, also plays an essential role in the development and progression of cancer has been one of the most significant breakthroughs in cancer biology over the last two decades (4).

Among the various types of epigenetic modifications of the genome, DNA methylation is the first one to be identified where the 5th carbon of the cytosine residue at CpG dinucleotides is methylated by the action of writer molecules known as DNA methyltransferases (5). Recent studies have revealed that methylation can take place beyond the CpG context. For example, in the brain and embryonic stem cells methylation can take place in non-CpG sites (CpH where H = A/T/C) (6, 7). However, methylation at non-CpG sites is not as frequent as CpG methylation. The distribution of methylation across the genome is cell type-specific and dynamic (8). Recent studies have demonstrated that DNA methylation-mediated transcriptional regulation is context dependent where methylation at the promoter region is typically related to repression of gene expression and methylation at the gene bodies is associated with activation of gene expression (9).

DNA methylation at the regulatory region of the gene can either directly hinder transcription factor binding and thereby cause transcriptional repression (10), or recruit “reader” molecules known as methyl-binding proteins (MBPs) at the methylated site which can then attract different members of the chromatin remodeling complex to cause transcriptional activation/repression depending on the cellular context (9, 11). However, the mechanisms by which the DNA methylation signatures are “read” and interpreted in different genomic context is not fully elucidated and may provide valuable information on how the gene expression programs are controlled in normal biological processes as well as in pathological conditions like cancer. The role of methyl-CpG-binding domain (MBD) proteins, that belongs to one of the three main families of MBPs (classification given in the next section), in the pathophysiology of different diseases including cancer has most recently been reviewed by Du et al. (12). On the other hand, the role of all the three known families of MBPs in relation to cancer was last reviewed by Parry and Clarke (13). However, since then, the field has gained newer insights into the functions and therapeutic targeting of different MBPs in cancer. In addition, several new proteins have been discovered to have methyl binding activities which warranted an update in the classification of MBPs. This review focuses on the known families of DNA methylation readers or MBPs and provides an updated summary of their roles in the context of cancer.

## Classification Of Mbps

Even though the existence of DNA methylation was first described in the late 1940s (14), it was not until the next 30 years when the importance of the process started to get appreciated due to the findings that revealed its role in the regulation of gene expression and cellular differentiation (15, 16). Several years after that two proteins with methyl binding activities were reported in mammals which were termed as methyl-CpG binding protein 1 (MeCP1) and MeCP2 (17, 18). However, later studies have demonstrated that MeCP1 is, in fact, a complex containing multiple proteins involved in chromatin remodeling (19–21). Therefore, MeCP2 is regarded as the first ever single MBP to be identified (17).

**Figure 1:**
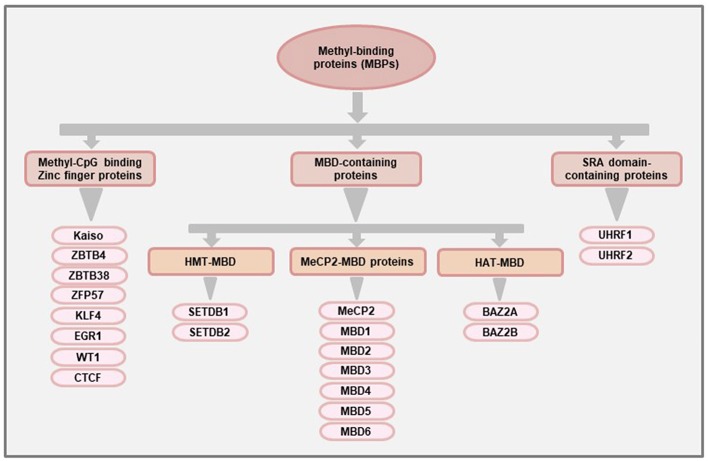
Classification of methyl-binding proteins (MBPs). The proteins with methyl-CpG binding abilities are broadly classified into three families based on the functional domains used for binding to methylated DNA. The “MBD-containing proteins” were the first group of MBPs to be identified and are further classified into three subfamilies (MeCP2-MBD, HMT-MBD, and HAT-MBD) based on the presence of functional domains other than MBD. The members of the HMT-MBD and HAT-MBD subfamilies have protein methyltransferase and acetylase activities respectively. The “Methyl-CpG binding Zinc finger proteins” have at least 8 members (Kaiso, ZBTB4, ZBTB38, ZFP57, KLF4, EGR1, WT1, CTCF) that can bind to methylated region using the Zinc finger motifs while the third family of MBPs consisting of UHRF1 and UHRF2 proteins uses their Set and RING-associated (SRA) domain to bind methylated DNA.

At the structural level, MeCP2 contains a MBD domain comprising 70–85 amino acids that can recognize and bind to methylated CpGs (22). The MBD domain was later used to identify other proteins with methyl-binding potentials (23). At present, there are 11 known proteins with MBD domains which are classified as the family of “MBD-containing proteins.” More than a decade after the discovery of MeCP2, a second family of MBPs were identified that recognizes the methylated DNA using the Zinc finger motifs. Hence, they are called the “Methyl-CpG binding zinc fingers” (24). This particular family has seen the most rapid expansion over the last few years and, at present, there are at least 8 members in this family (25). The third family of MBPs was identified based on their ability to bind methylated DNA using the Set and RING-associated (SRA) domain and hence called the “SRA domain-containing proteins”. A schematic classification of the three main families of MBPs is shown in Figure 1, and the general and pathophysiological functions (in cancer) of each of the proteins is summarized in Table 1.

**Table 1:** Summarized features and functions of different MBPs.

**Name of protein**	**General feature and function**	**Type of cancer**	**Role in cancer**	**Reference**
MeCP2	It is the founding member of MBPs and acts as a transcriptional repressor. It is a 50-kDa protein encoded by a gene on the X chromosome and functions in nuclear organization, chromatin compaction and fiber binding, chromatin looping, rearrangement of heterochromatin, regulation of splicing	Prostate cancer	Loss of function of the gene decreased cell proliferation and increased apoptosis	(26, 27)
		Breast cancer	Its expression showed strong association with estrogen receptor status	(28)
		Gastric cancer	Elevated expression showed association with shorted patient survival	(29)
		Liver cancer	Promotes cell proliferation through the activation of ERK1/2	(30)
MBD1	It is a multidomain protein encoded by a gene located on chromosome 18. The main function of MBD1 is to cause transcriptional repression through methylation of H3K9 and heterochromatin formation.	Colorectal cancer	Acts as a tumor suppressor gene and the mRNA expression is downregulated with disease progression	(31)
		Pancreatic cancer	Overexpression of MBD1 showed an association with lymph node metastasis	(32)
		Lung cancer	Polymorphisms in MBD1 gene associated with lung cancer development	(33)
		Prostate cancer	*MBD1* depletion promotes cell invasion; the protein expression gradually decreases with the increase in cancer grade.	(27, 34)
MBD2	It is a multiexon gene located on chromosome 18. It can function as a transcriptional repressor and activator depending on the cellular context. It also plays role in mediating immune response.	Colorectal cancer	*Mbd2*-deficient mice are resistant to intestinal cancer	(35)
		Breast cancer	Plays a role in tumor progression and proliferation. Knockdown of *MBD2* activation the expression of tumor suppressors genes like *DAPK1* and *KLK10*	(36)
		Colon cancer	Represses the expression of tumor suppressor genes (*p16/Ink4A, p14/ARF*)	(37)
		Liver cancer	Mediates silencing of the tumor suppressor glutathione S-transferase gene (*GSTP1*)	(38)
		Bladder cancer	Higher expression of *MBD2* shows associations with reduced risk of tumorigenesis	(39)
		Prostate cancer	Represses the expression of key tumor suppressor gene	(40)
MBD3	It is an encoded multiexon gene located on chromosome 19 and interacts with NuRD complex to cause transcriptional repression.	Liver cancer	MBD3 inhibits formation of cancer stem cells	(41)
		Pancreatic cancer	Decreased expression in patients correlates with poor survival; MBD3 overexpression inhibits migration and invasion	(42)
		Malignant glioma	Decreased expression in patients correlates with reduced with decreased overall survival and progression-free survival	(43)
MBD4	The *MBD4* gene is located on chromosome 3. The encoded protein has DNA glycosylase activity and functions in DNA repair.	Colorectal cancer	Mutation in the *MBD4* gene triggers mismatch repair deficiency	(44)
		Acute myeloid leukemias (AMLs)	Germ line *MBD4* deficiency predisposes to AML	(45)
		Gastric cancer	Frameshift mutation of *MBD4* triggers DNA mismatch repair deficiency which induce cancer progression	(46)
MBD5	It is a 159-kD multidomain protein encoded by a gene located on chromosome 2. The protein can bind to mammalian polycomb repressive complex PR-DUB; but cannot bind to methylated DNA even though it contains the MBD domain. It plays a role in development.	-	Not known yet	-
MBD6	It is a 101-kD multidomain protein that binds to mammalian polycomb repressive complex PR-DUB; but cannot bind to methylated DNA even though it contains the MBD domain. It has a role in maintaining cellular stemness.	Gastric and colorectal cancer	Mutation and abnormal expression of *MBD6* was reported in patients	(47)
SETDB1 (Also known as KMT1E or ESET)	It is a 143-kD multidomain protein encoded from a gene located on chromosome 1. Plays a role in transcriptional repression by forming heterochromatin.	Colorectal cancer	Higher SETDB1 expression showed inverse correlation with patient survival rate	(48)
		Sporadic cutaneous melanoma	Higher expression showed association with several prognostic parameters in melanoma	(49)
		Liver cancer	Elevated expression showed association with disease progression, increased metastasis, and poor prognosis of patients	(50)
		Breast cancer	Knockdown of SETDB1 cell proliferation, migration, and cell cycle	(51)
SETDB2 (also known as CLLD8)	It is a 81-kD multidomain protein that has protein methyltransferase activity, and plays role in transcriptional repression by forming heterochromatin	Gastric cancer	Elevated expression correlated with disease progression	(52)
BAZ2A (Also known as TIP5)	The *BAZ2A* gene is located on chromosome 12 and the encoded protein plays role in chromatin remodeling and subsequent regulation of gene expression.	Prostate cancer	Overexpression is an individual biomarker to predict disease recurrence	(53)
BAZ2B	The *BAZ2B* gene is located on chromosome 2, and the encoded protein can bind to histones and chromatin remodeling complexes via the PHD and bromodomains.	-	Not known yet	-
Kaiso	It is encoded by ZBTB33 gene located on X chromosome. It plays role in cell adhesion and signaling in the cytoplasmic compartment while it acts as a transcriptional repressor in nucleus.	Prostate cancer	Promotes cell migration and invasiveness via regulating miR-31 expression	(54)
		Colon cancer	Kaiso depletion induced tumor suppressor gene expression. In addition, the cancer cells became susceptible to cell cycle arrest and cell death	(55)
		Intestinal cancer	Deficiency decreases tumor size and increases life span of Apc (Min/+) mice	(56)
		Lung cancer	Associated with poor prognosis	(57)
		Breast cancer	Depletion of the protein reduced cancer cell proliferation, invasion and metastasis	(58, 59)
ZBTB4	It is encoded by ZBTB4 gene located on chromosome 17. It plays a role in cell cycle and acts as a transcriptional repressor of several oncogenic genes.	Breast cancer	Expression is positively correlated with relapse-free survival	(60)
		Neuroblastoma	ZBTB4 depletion arrests cell cycle and promotes cancer cell survival by suppressing apoptosis	(61)
		Prostate cancer	Elevated expression may serve as a prognostic factor for longer patient survival.	(62)
		Skin cancer	ZBTB4-deficient mice are susceptible to developing carcinogen-induced cancer.	(63)
ZBTB38	It is encoded by ZBTB4 gene located on chromosome 3. It plays a role in cell proliferation, differentiation, DNA replication and transcription	Prostate cancer	Polymorphisms in ZBTB38 increase prostate cancer risk	(64)
		Bladder cancer	ZBTB increases cell invasion, migration, and metastasis	(65)
ZFP57	It is encoded by ZFP57 gene located on chromosome 6. It plays a role in genomic imprinting and regulation of gene expression	Glioblastoma	Elevated expression of the gene has been found in patients with high-grade glioblastoma	(66)
		Lung cancer	Has been identified as a disease susceptibility locus for the development of lung cancer	(67)
KLF4	It is encoded by KLF4 gene located on chromosome 9. It plays a role in cell proliferation, differentiation, DNA damage response, cell cycle, and apoptosis.	Gastric cancer	Lower level of KLF4 showed association with poor survival	(68)
		Colorectal cancer	Functions as a tumor suppressor	(69)
		Bladder cancer	KLF4 expression is downregulated in both cell lines and patient tissues; restoration of KLF4 gene decreased cell proliferation and increased apoptosis	(70)
		Breast cancer	Both RNA and protein expression are increased during the progression of breast tumor. KLF4 knockdown reduces cell migration, invasion and colony formation	(71, 72)
		Glioblastoma	Promotes cell adhesion and migration	(73)
		Skin cancer	Elevated expressed showed association with cancer progression and metastasis	(74)
EGR1	It is encoded by EGR1 gene located on chromosome 5. It plays a role in the maintenance of synaptic plasticity, cell proliferation, differentiation, cell cycle, apoptosis, wound healing, and regulation of gene expression.	Prostate cancer	Overexpressed in prostate cancer	(75, 76)
		Wilms' tumor	Overexpression of the gene enhances tumorigenicity *in vivo*	(77)
		Breast cancer	It is downregulated in breast tumors. Moreover, overexpression of EGR1 inhibits cell proliferation and blocks cell cycle at G0/G1 phase	(78, 79)
		Glioblastoma	EGR1 expression is lower in glioma tissue compared to normal brain tissues and knockdown of the gene decreased cell proliferation and tumorigenesis both *in vitro* and *in vivo*	(80)
		Fibrosarcoma	It suppresses fibrosarcoma cell growth *in vitro* through the induction of TGF-β1	(81)
		Lung cancer	Functions as a tumor suppressor by enhancing the KRT18 expression	(82)
WT1	The WT1 gene, located in chromosome 11, encodes for the protein that functions in cell growth, proliferation, differentiation, cell cycle, maintenance of genomic stability, and regulation of gene transcription.	Wilms' cancer	Expression is downregulated in this pediatric cancer; mutations in the WT1 gene showed association with the occurrence of the sporadic form of the disease	(83, 84)
		Breast cancer	Higher expression of the WT1 gene showed association with poor prognosis in patients	(85)
		Leukemia	Elevated expression of the gene showed association with poor patient outcome	(86)
		Head and neck cancer	Increased expression of the gene showed correlation with higher tumor stage	(87)
		Ovarian cancer	Protein expression increased in patients with cancer and showed indication of unfavorable prognosis	(88, 89)
UHRF1 (Also known as ICBP90 in human and Np95 in mouse)	It is encoded from a gene located on chromosome 19. Plays a role in regulation of cell proliferation, cell cycle, apoptosis, as well as in DNA repair. It also plays a crucial role in the maintenance of DNA methylation in daughter strands; links DNA methylation to histone modification.	Breast cancer	Promotes cell proliferation and migration	(90)
		Pancreatic cancer	Promotes the growth, migration, and metastasis	(91)
		Colorectal cancer	Promotes CRC growth and metastasis through the repression of p16 (ink4a)	(92)
		Lung cancer	Involved in the silencing of tumor suppressor genes	(93)
		Hepatocellular carcinoma	Elevated UHRF1 is associated with poor prognosis	(94)
		Gastric cancer	Promotes invasion and metastasis, downregulates tumor suppressor genes	(95)
		Ovarian cancer	Depletion of UHRF1 decreased proliferation and induce apoptosis	(96)
		Prostate cancer	Downregulates the expression of tumor suppressor genes	(97)
UHRF2 (Also known as NIRF or Np97)	It is encoded from a gene located on chromosome 9. Plays role in regulation of cell proliferation, cell cycle, apoptosis. It can read 5 hmC, 5 mC on DNA as well as H3K9 methylation.	Colon cancer	Involved in invasion and metastasis	(98)
		Intrahepatic Cholangiocarcinoma	Involved in cell proliferation, invasion, migration, and decreases apoptosis	(99)
		Breast cancer	Involved in cell proliferation	(100)
		Osteosarcoma	Interacts with E2F1 to induce apoptotic cell death	(101)

### MBD-Containing Proteins

**Figure 2:**
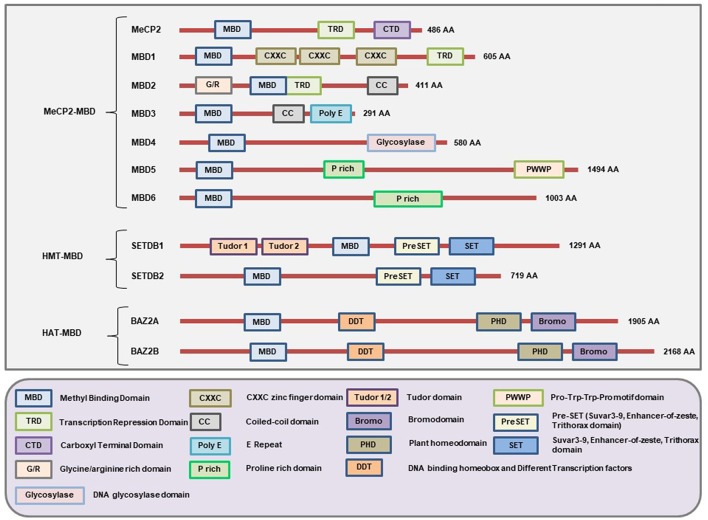
Schematic representation of the major domains present in different MBD-containing proteins. All members of this family of proteins have the MBD domain. However, not all 11 members under this family can bind methylated DNA but were classically grouped under the same family because of having the MBD domain. Each member of this family has domains other than MBD that gives them unique characteristics to carry out different cellular functionalities. The MeCP2, MBD1, and MBD2 contain a TRD domain that helps them to recruit chromatin remodeling corepressors and thereby cause transcriptional silencing. In addition, the MBD1 protein may have two or three CXXC-domains due to splicing of its gene. The first two CXXC domains of MBD1 bind to methylated DNA while the third CXXC domain binds to unmethylated DNA. MBD2 has unique G/R rich domain that allows protein-protein interaction as well as post-translational modification. Due to a mutation in the region of the gene encoding the MBD domain of MBD3, it cannot bind to methylated DNA. However, MBD3 can interact with other chromatin remodeling complex and play a role in the regulation of gene expression. The glycosylase domain at the C-terminal end of MBD4 provides it with the unique function of having DNA glycosylase activity while the N-terminal MBD helps it to bind methylated DNA. MBD5 and MBD6 cannot bind to methylated DNA but can interact with the mammalian polycomb deubiquitinase complex PR-DUB. In addition, the PWWP (Pro-Try-Try-Pro) motif of MBD5 helps it bind to the methylated histones. The SET domain provides SETDB1 and SETDB2 the ability to act as protein methyltransferase and the Tudor domains help SETDB1 to bind to methylated histones. In addition, these proteins also have PreSET domain located N-terminus to the SET domain that functions in stabilizing the SET domain. Both BAZ2A and BAZ2B contains a PHD domain to bind unmodified histone while bromodomain and DDT domains allow them to recognize the acetylated histones and DNA binding abilities respectively. (Figure not drawn to the exact scale of the proteins).

This is the first family of MBPs to be identified. All members of this family have the conserved MBD domain (NCBI Conserved Domain Database ID: cl00110 and cd00122). However, they also have some additional domains which provide them with specific features (Figure 2) and functions (Table 1). Based on the presence of domains other than MBD, the members of this family are further classified into three categories: (1) MeCP2-MBD, (2) HMT-MBD, and (3) HAT-MBD. The three subfamilies of MBD-containing proteins are phylogenetically distinct from each other (102). In addition, not all the proteins can bind to methylated DNA but are historically classified together based on their structural features rather than methyl-binding abilities.

#### MeCP2

The MeCP2 is a 50-kD multidomain transcriptional repressor protein encoded from an X chromosome gene containing four exons that give rise to two isoforms *MECP2_e1* and *MeCP2_e2* depending on the presence or absence of the second exon (103). These isoforms provide different functionalities to each of these proteins.

The MBD domain of MeCP2 can bind to a single methylated CpG. However, the binding of MBD requires an A/T-rich sequence near the methylated CpG regions (104). Apart from the MBD domain, MeCP2 also has the transcription repression domain (TRD) involved in mediating gene silencing through the recruitment of chromatin remodeling complex comprising the Sin3A co-repressor and histone deacetylases (HDAC1 and HDAC2) (11) (Figure 2). Some of the other functions of the MeCP2 protein include nuclear organization, chromatin compaction and fiber binding, chromatin looping, rearrangement of heterochromatin, regulation of splicing, and DNA methylation (105). Mutations of the *MECP2* gene may cause a severe neurodegenerative disease known as the Rett syndrome that mainly affects females due to the presence of the gene on the X chromosome (106).

MeCP2 is also involved in cancer through it's binding to the hypermethylated regions of promoters of tumor suppressor genes and thereby cause their subsequent repression in breast cancer (107), prostate cancer (108), lung cancer (109), liver cancer (110), and colorectal cancer (111). Elevated expression of the *MeCP2* gene has been reported in different cancers (28, 29, 112). In the case of liver cancer cells, MeCP2 promotes proliferation via the activation of ERK1/2 with simultaneous inhibition of p38 activity (30). Increased MeCP2 expression has shown an association with shorter overall survival and disease-free survival of gastric cancer patients (29). Loss of function of *MeCP2* has been reported to inhibit cell proliferation and increased apoptosis of prostate cancer cells *in vitro* (26, 27). In addition, treatment with several natural compounds has shown to downregulate the elevated MeCP2 expression in prostate and breast cancer cells *in vitro* (113, 114).

#### MBD1

MBD1 is a multidomain protein consisting of an N-terminal MBD domain that can bind methylated DNA, a C-terminal TRD domain that can mediate protein-protein interaction, and two or three CXXC-type Zinc fingers in between MBD and TRD domains (103). It is encoded from a multiexon gene located on chromosome 18. MBD1 has 13 isoforms due to alternative splicing of the gene (13). The major distinction between the encoded proteins from the differentially spliced isoforms is the retention of two or three CXXC-type Zinc finger motifs (115). The isoforms having the first two CXXC-type Zinc finger motifs can bind to methylated CpG to cause transcriptional repression, whereas the isoforms having the third CXXC-type Zinc finger motif has the ability to bind unmethylated DNA (115, 116). This implies that the isoforms having all three CXXC-type Zinc finger motifs can cause transcriptional repression regardless of the DNA methylation status.

MBD1 interacts with MBD1-containing chromatin-associated factor 1 (MCAF1) and histone methyltransferase SETDB1 to form MBD1:SETDB1:MCAF1 complex that methylates lysine residue 9 of histone H3 to promote heterochromatin formation (117–119). MBD1 can also interact with another histone methyltransferase known as Suv39h and cause H3K9 methylation. *Mbd1* knockout mice (*Mbd1*^−/−^) are viable and healthy but show some defects during neural stem cell differentiation along with increased chromosome instability (120).

MBD1 plays a role in tumorigenesis by repressing tumor suppressor genes like *CDH1, RASSF1A, TIMP3, P14ARF*, and *Rb* (121). In human pancreatic carcinomas, elevated expression of MBD1 showed association with lymph node metastasis (32). Furthermore, knockdown of *MBD1* inhibited cell proliferation, invasion, and increased apoptosis of pancreatic cancer cells (122, 123). However, the oncogenic role of MBD1 is not typical for all cancers. For example, MBD1 may also act as a tumor suppressor in colorectal cancer (31). A hypermethylation-mediated downregulation of the *MBD1* gene is seen along with the progression of metastatic colorectal cancer (31). Moreover, polymorphisms in the *MBD1* gene have shown an association with the increased risk of developing lung cancer (33). In prostate cancer cells, depletion of *MBD1* increased cell invasion with no change in apoptosis compared to control cells where *MBD1* expression was intact (27). Moreover, analysis of patient biopsies revealed that the MBD1 protein expression gradually decreased with the increase of prostate cancer grade (34). Taken together, these observations indicate a dual role of MBD1 both as a tumor suppressor and oncogene depending on the type of cancer.

#### MBD2

The *MBD2* is a multiexon gene located on chromosome 18 of both human and mouse genomes. The encoded protein (MBD2) from this locus shows more than 70% amino acid sequence similarity with another protein called MBD3 (23). In addition, a high level of gene-sequence homology exists between human and mouse *MBD2* and *MBD3* genes which is suggestive a gene duplication event during the course of evolution (102). Initial studies proposed that MBD2 functions as a transcriptional repressor by recruiting co-repressors like the NuRD (Nucleosome Remodeling Deacetylase) complex to the methylated sites (19).

**Figure 3:**
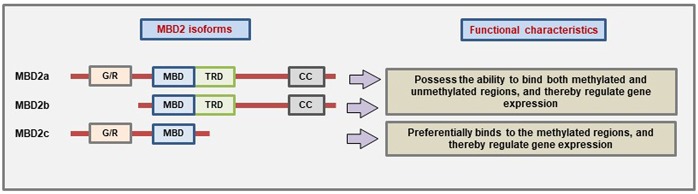
Characteristic domain architecture and function of different MBD2 isoforms. MBD2a is the canonical isoform containing four domains while MBD2b lacks the N-terminal G/R-repeat due to the use of alternative start site during translation. On the other hand, the MBD2c isoform is formed due to the inclusion of an alternative third exon which produces a premature stop codon, and as a result, the MBD2c lacks the TRD and CC domains (Figure not drawn to the exact scale of the proteins).

MBD2 protein has three main isoforms due to the use of alternative translational start site and alternative splicing: MBD2a, MBD2b, and MBD2c (also called MBD2t) (23, 124). The presence of different domains which give the different MBD2 isoforms the ability to interact with different binding partners and thereby carry out different functions (Figure 3). However, all three isoforms have the MBD domain to bind to methylated CpG. MBD2a is the canonical isoform and contains an N-terminal glycine-arginine (GR) repeat that can undergo post-translational modification, followed by the MBD domain, the TRD domain, and lastly the coiled-coil (CC) domain at the c-terminal region that has the ability to mediate protein-protein interactions (12, 125). The MBD2b uses an alternative start site during translation, and the only difference from MBD2a is the absence of the N-terminal GR repeat. The presence of the MBD and TRD domain in both of these isoforms helps them to bind different corepressor complexes to mediate transcriptional repression (126). The third isoform MBD2c is devoid of the TRD and CC domains due to the inclusion of an alternative exon 3 that produces a truncated protein (23). The MBD2c may function differently than the other isoforms. For example, in human pluripotent stem cells (hPSC), MBD2a interacts with NuRD to promote cell differentiation while MBD2c mediates the reprogramming to pluripotency (127).

It has been shown that MBD2 can also bind to unmethylated DNA to cause changes in gene expression (128). However, the TRD-domain deficient MBD2c isoform cannot bind to unmethylated DNA which suggests that MBD2 binding to the unmethylated regions of the DNA is dependent on the interaction between TRD domain and NuRD complex (128). More recent evidence suggests that the MBD2 protein can also mediate the activation of gene expression (129–131). MBD2 has been proposed to function as a demethylase enzyme that can remove or “erase” the DNA methylation marks (132). However, this finding has been contested by several others (126, 133). The *Mbd2* knockout mice (*Mbd2*^−/−^) are viable and do not show any abnormalities during embryonic development even though the female *Mbd2*^−/−^ mice have been reported to show some abnormalities related to maternal behavior (134).

MBD2 plays an important role in cancer by silencing key tumor suppressor genes in prostate cancer (40), colon cancer (37), and liver cancer (38). On the other hand, in several cancer-types, MBD2 has been shown to mediate transcriptional repression of human telomerase reverse transcriptase (*hTERT*) which is suggestive of a tumor suppressive function of MBD2 (135). In breast cancer, Müller et al. could not detect any discernable difference in *MBD2* expression (28), while Billard et al. detected a statistically significant upregulation of *MBD2* in the mammary tumor (136).

Stable knockdown of the *MBD2* gene suppressed the proliferation of several breast cancer cell lines *in vitro* and decreased tumor volume *in vivo* (36). Furthermore, it was demonstrated that tumor suppressor genes like *DAPK1* and *KLK10* are de-repressed upon depletion of MBD2 in breast cancer cells. Interestingly, MBD2 has been also shown to function in the maintenance and spread of DNA methylation at specific regulatory regions of prostate cancer cells (137). Moreover, knockout of *Mbd2* gene mice protected against tumorigenesis when crossed with *Ap*c^*Min*/+^ mice (a rodent model for colorectal cancer) (35). The loss of MBD2 function causes downregulation of the Wnt signaling pathway which plays a major role in the development of colorectal cancer (35, 138). However, later studies have found that depletion of several other epigenetic and chromatin binding factors like Kaiso, DNMTs, and Brg1 also downregulated the Wnt signaling pathway and thereby protected from tumorigenesis (56, 139, 140). This suggests that the downregulation of Wnt signaling is not MBD2-specific, and it is instead a result of a general perturbation of chromatin remodeling complex (8).

Emerging evidence supports that MBD2 plays a role in immunity partly because of its tissue localization pattern. Among the various members of the MeCP2-MBD family, MBD2 shows the highest expression in spleen which is a major site for both adaptive and innate immune responses (8, 141). Wang *et al*. demonstrated that MBD2 regulates the expression of Foxp3 which is the master regulator of regulatory T cells (Tregs) (129). They have shown that MBD2 binds to the Treg-specific demethylation region (TSDR) located upstream of the *Foxp3* gene and thereby facilitates TET2-mediated demethylation to induce *Foxp3* expression. Furthermore, knockout of *Mbd2* gene reduced the number of Tregs and impaired the immunosuppressive function meditated by the Tregs. Interestingly, the *Mbd2*^−/−^ mice did not develop autoimmunity which makes it an attractive target in pathological conditions like cancer where the Tregs have been suggested as potential targets for immunotherapy (142).

Even though MBD2 is an attractive anti-cancer target, drugs that can specifically target MBD2 have not been identified to date. Our group has previously shown that the treatment of cancer cells with the universal methyl group donor S-adenosylmethionine (SAM) downregulates *MBD2* expression and shows anti-proliferative and anti-metastatic effects both *in vitro* and *in vivo* (143, 144). Moreover, antisense oligonucleotides against *MBD2* gene also showed promising anti-cancer effects in xenograft models (145). However, caution must be taken while targeting MBD2 to avoid any potential negative ramification (13).

#### MBD3

MBD3 is encoded from a multiexon gene located on chromosome 19 that produces three isoforms: MBD3a, MBD3b, and MBD3c (23, 146). As mentioned before, MBD3 has a high degree of protein sequence similarity with MBD2 but lacks the GR-rich domain found in the full-length canonical isoform of MBD2a. Due to the presence of a point mutation in the region coding for the MBD domain, the selective binding affinity for methylated CpG is abolished in case of MBD3 protein (8, 147). However, MBD3 has been shown to preferentially bind to 5-hydroxymethylated (5 hmC) CpGs *in vitro* (148). Furthermore, MBD3 colocalizes with the DNA demethylase TET1 *in vivo* and is required maintenance of 5 hmC (148). MBD3 is a component of NuRD repressive complex and causes a transcriptional repression of genes (149). The association of MBD3 and MBD2 with NuRD complex is mutually exclusive which is indicative of different functional properties (149). The MBD3-NuRD complex plays a role in pluripotency and differentiation of embryonic stem cells (146, 148). Moreover, knockout of *Mbd3* gene is embryonic lethal (134).

MBD3 has been shown to function as a tumor suppressor. In clinical specimens obtained from pancreatic cancer patients, decreased *MBD3* gene expression showed an association with poor patient survival (42). A similar observation was noted in malignant glioma where patient samples with reduced MBD3 expression showed a correlation with decreased overall survival and progression-free survival (43). Moreover, knockdown of *MBD3* gene promoted cancer cell migration and invasion while overexpression inhibited those effects (42). In liver cancer, the MBD3-NuRD complex inhibited the induction of cancer stem cells (CSC) whereas knockdown of *MBD3* upregulated the expression of CSC-related genes (41). In addition, knockdown of *MBD3* also increased the c-Jun protein expression (41). Other studies have shown that N-terminal phosphorylation of c-Jun inhibits the recruitment of MBD3-NuRD complex and thereby causes the upregulation of tumorigenesis promoting genes (150). While anti-cancer targeting of c-Jun may serve as a viable and attractive strategy to induce MBD3 expression, further research is needed in this regard.

#### MBD4

MBD4 is encoded from a gene located on chromosome 3 and functions in DNA repair mechanism. The N-terminal MBD domain allows it to bind to the methylated DNA. However, the unique feature of the protein lies at the C-terminal glycosylase domain that makes it the only MBP to have DNA glycosylase activity to repair mismatches of hypermutable CpG (151). Spontaneous deamination of 5-methylcytosine (5 mC) is one of the primary sources of somatic mutation that the body accumulates with time (45), and MBD4 protects from such mutations by interacting with mutL homolog 1 (MLH1) to trigger the DNA repair mechanism (152).

The homozygous *Mbd4* gene knockout mice are viable and do not show any defective phenotype (153). When these knockout mice were crossed with the rodent model of colorectal cancer (*Ap*c^*Min*/+^ mice), there was an increase in tumorigenesis (153). Mutations in the *MBD4* gene has also been observed in human colorectal and gastric cancer patients that trigger mismatch repair deficiency (44, 46). A recent study has shown that germline mutation of *MBD4* increases the susceptibility to develop acute myeloid leukemia (AML) (45). Taken together these studies support a tumor suppressive function of MBD4 in cancer even though more research is needed to elucidate whether this effect is common for all cancer or specific for certain types of cancer.

#### MBD5 and MBD6

The MBD5 and MBD6 proteins are encoded from their respective genes located on the chromosomes 2 and 6. They were initially identified through protein homology search and classified as MBD proteins due to the presence of the MBD domain (154). They still remain the least functionally characterized members of the MeCP2-MBD subfamily of proteins. It has been shown that both MBD5 and MBD6 localize to the heterochromatin *in vitro* and cannot bind to the methylated DNA (155). Both these proteins contain a proline-rich domain that can mediate protein-protein interactions during intracellular signaling. In addition, MBD5 has a C-terminal PWWP (Pro-Try-Try-Pro) motif that may bind to the methylated histones.

MBD5 and MBD6 can both interact with the mammalian polycomb repressive complex PR-DUB that can cause deubiquitination of different cellular targets (156). They are highly expressed in the brain and testis, which is suggestive of their possible role in development (155, 157, 158). Indeed, abnormalities in the *MBD5* gene has been reported in patients with mental retardation (159, 160). Moreover, the *Mbd5* knockout mice showed deficiencies in postnatal growth and aberrations in glucose homeostasis (161). The MBD6 protein has been reported to be involved in cellular stemness by regulating the activity of Oct4 (octamer-binding transcription factor 4) (162). Whether these proteins have any role in cancer, is still not clear. Mutation and abnormal expression of the *MBD6* gene have been reported in gastric and colorectal cancers (47). Further studies are needed to know whether these genes are implicated in cancer.

### HMT-MBD

This subgroup of MBD-containing proteins consists of two members: SET domain bifurcated 1, SETDB1 (also known as KMT1E or ESET) and SETDB2 (also known as CLLD8). Apart from having the MBD domain, these proteins also have the SET (SuVar3-9, enhancer of Zeste, Trithorax) domain for interacting with other proteins (Figure 2). Both SETDB1 and SETDB2 have the protein lysine methyltransferase activity which enables them to cause transcriptional repression (163, 164). The exact molecular mechanism on how SETDB2 mediates gene repression is still not clear (52). However, the mechanism of SETDB1 mediated transcriptional repression is known. Briefly, the SETDB1 protein interacts with MBD1 which directs it to the methylated CpG region. Then SETDB1, through its methyltransferase activity, mediates trimethylation of histone H3 on lysine 9 (H3K9me3) to cause heterochromatin formation and thereby repress transcription. Both SETDB1 and SETDB2 are involved in embryonic development (165, 166).

Emerging evidence supports that SETDB1 functions as an oncogene in many cancers that include sporadic cutaneous melanoma (49), liver cancer (50), and colorectal cancer (48). It has been shown that SETDB1 can directly interact with the *de novo* DNA methyltransferase DNMT3A in order to attach at the promoter region and mediate the transcriptional silencing of well-known tumor suppressor genes *RASSF1A* (in breast cancer cells) and *P53BP2* (in cervical cancer cells) (167). Moreover, knockdown of *SETDB1* led to the suppression of breast cancer proliferation and migration *in vitro*, and also inhibited tumorigenesis *in vivo* (51).

The gene encoding for the SETDB2 protein was initially identified as a potential candidate for leukemogenesis (168). Elevated expression of SETDB2 has shown association with gastric cancer progression (52). Further studies are warranted to know whether the oncogenic characteristics of SETDB1 and SETDB2 are universal for all cancers or just specific to particular cancer type.

### HAT-MBD

This subgroup also consists of two members: BAZ2A (bromodomain adjacent to zinc finger domain 2A, also known as TIP5) and BAZ2B. Apart from the MBD domain, they also contain the DDT (DNA binding homeobox and Different Transcription factors), PHD (Plant homeodomain), and bromodomains (Figure 2). The PHD domain helps to bind to the unmodified histone, and the bromodomain helps to recognize the acetylated lysine residues of a protein (169–171). These proteins have histone acetyltransferase activities and are involved in chromatin remodeling (13). The MBD domain of these proteins cannot bind to the oligonucleotides that are methylated (13). However, the MBD domain of BAZ2A binds to unmethylated DNA *in vitro* (172).

Not much is known about the involvement of these proteins in cancer. The BAZ2A protein showed an association with the epigenetic alteration in prostate cancer patients, and its overexpression has been suggested to be an individual biomarker to predict disease recurrence (53). Further studies are needed to know the exact roles of these proteins in cancer.

### Methyl-CpG Binding Zinc Finger Proteins

**Figure 4:**
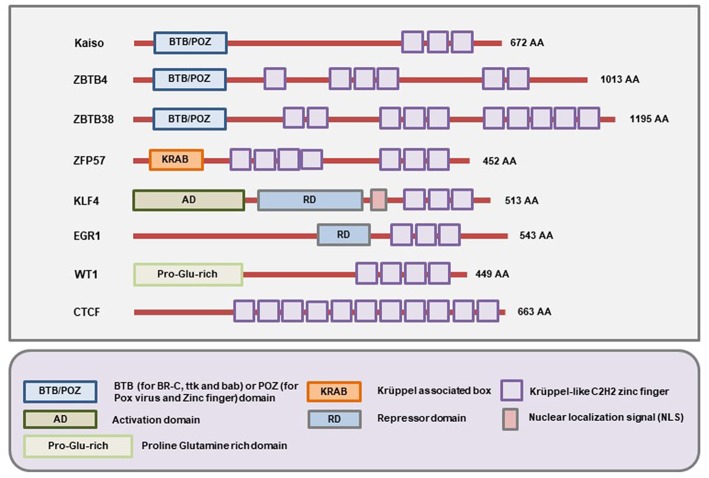
Schematic representation of the domains present in different Methyl-CpG Binding Zinc finger proteins. All members of this family contain C-terminal Zinc finger motifs that allow them to bind to both methylated and unmethylated DNA. In addition, Kaiso, ZBTB4, and ZBTB38 contain the BTB/POZ domain while ZFP57, WT1, EGR1 contain the Krüppel associated box (KRAB), Proline-Glutamine rich (Pro-Glu-rich), and repressor domains (RD) respectively. On the other hand, the KLF4 protein contains an activation domain (AD), repressor domain (RD), and an NLS (nuclear localization signal/sequence) apart from the Zinc fingers (Figure not drawn to the exact scale of the proteins).

The members of this family of MBPs have Zinc finger motifs at the C-terminal region which allow them to bind both methylated and unmethylated DNA. The initially identified member of the family is Kaiso whose methylated DNA-binding ability was demonstrated by the labs of Egor Prokhortchouk and Adrian Bird in 2001 (24). Later on, two homologs of the Kaiso proteins, Zinc-finger and BTB domain containing 4 (ZBTB4) and ZBTB38 were discovered (173). Like Kaiso, ZBTB4 and ZBTB38 both have the N-terminal broad-complex, tramtrack, and bric-a-brac/poxvirus and zinc finger (BTB/POZ) domain (Figure 4). For several years, this branch of MBPs only comprised of these three known members. More recently, several other members (ZFP57, KLF4, WT1, EGR1, and CTCF) of this branch have been identified (25). These proteins have the Zinc finger motifs in the C-terminal region for binding to the methylated DNA. However, the N-terminal domain is not similar to Kaiso, ZBTB4, and ZBTB38 (Figure 4). So, according to the current literature, there are at least 8 members in this family of MBPs (25).

#### Kaiso

Kaiso is a transcription factor that was initially identified as the cytoplasmic binding partner of p120-catenin (174). It is encoded by *ZBTB33* gene located on the X chromosome of the human genome (175). Its ability to bind a pair of methylated CpG dinucleotides was demonstrated experimentally (24). Kaiso can also bind to unmethylated DNA at Kaiso binding sites (KBS) with a consensus sequence of TCCTGCNA, where N is any nucleotide (175). The Kaiso binding affinity at KBS is higher than that of methylated CpG dinucleotides. Qin et al. have further demonstrated that Kaiso cannot bind the hydroxymethylated CpG dinucleotides (176).

Kaiso plays roles both at the cell surface and the nucleus. At the cell surface, it plays roles in cell adhesion as well as signal transmission by regulating the stability of the cadherins (177). On the other hand, in the nucleus, it directs the Nuclear Receptor Corepressor Complex (N-CoR) to both methylated and unmethylated regions on the DNA and thereby promotes a repressive chromatin state (178). In general, Kaiso is considered as a transcriptional repressor even though it can also bind to the unmethylated regions of actively expressed genes (179). Kaiso also localizes to the centrosomes and mitotic spindles during cell cycle progression (180).

Kaiso expression and localization has shown association with different types of cancer that include lung (57), prostate (54), breast (58, 59), and colon (55). Kaiso causes transcriptional repression of several well-known tumor suppressor genes like Retinoblastoma (*Rb*), Hypermethylated In Cancer 1 (*HIC1*) and Cyclin-Dependent Kinase Inhibitor 1A (*CDKN1A*) (55, 181). Depletion of Kaiso inhibited breast cell proliferation and survival by increasing apoptotic cell death (58). Moreover, Kaiso-deficient mice crossed with *Ap*c^(*Min*/+)^ mice have shown lower susceptibility of developing intestinal tumors compared to the control mice where the gene encoding for the Kaiso protein was not knocked out (56).

Since Kaiso binds to both methylated and unmethylated DNA, it is difficult to understand how crucial a role its methylated CpG binding property plays in cancer. For example, Kaiso can cause transcriptional repression of unmethylated Matrix Metallopeptidase 7 (*MMP7*) and Cyclin D1 (*CCND1*) genes as well as methylated Metastasis Associated 1 family member 2 (*MTA2*) gene in lung cancer (182). In this case, all three genes have roles in cancer progression and, therefore, further research is needed to pinpoint the precise role of Kaiso's methylated CpG binding property in cancer.

Even though Kaiso's role as a potent repressor is now well-established and Kaiso-deficient mice are viable, therapeutic agents to inhibit Kaiso have not been developed so far. Through computer-aided drug designing methods, it has been shown that a natural compound (chem. ID 28127) can strongly inhibit Kaiso (183). However, detailed experimental evidence is needed in this regard.

#### ZBTB4

ZTBT4 can bind to a single methylated CpG which is different from Kaiso that needs two methylated CpG dinucleotides. Moreover, ZBTB4 can also bind to the unmethylated KBS (173). This makes ZBTB4 a bimodal protein that can bind to both methylated and unmethylated DNA. However, the ZBTB4 binding affinity for methylated CpG is higher than that of KBS. ZBTB4 can function as a transcriptional repressor by recruiting Sin3/histone deacetylases and thereby silence the expression of the target gene (61). Furthermore, ZBTB4 has been shown to localize at the highly methylated centromeric and pericentromeric repeats (173, 184). This localization is abolished in the cells that lack methylation of DNA.

Downregulation of ZBTB4 has been found in several common malignancies that include neuroblastoma (61), breast (60) and prostate cancer (62). Decreased levels of ZBTB4 in the tumor correlated with higher genomic instability (63). In breast cancer patients, ZBTB4 protein and mRNA are both downregulated, and its expression showed an association with relapse-free survival (60). Elevated levels of ZBTB4 also correlated with longer survival of prostate cancer patients (62). ZBTB4 is involved in the crosstalk between DNA methylation and histone modification. It has been shown that ZBTB4 causes transcriptional silencing of specificity protein (Sp) transcription factors (Sp1, Sp3, and Sp4) that are required for histone methyltransferase EZH2 expression (185). EZH2 is currently considered a target for anti-cancer therapy since it has a role in cell proliferation and cell division. ZBTB4 also causes repression of key angiogenesis factors, vascular endothelial growth factor (VEGF) and its receptor (VEGFR) (60). The *Zbtb4*-deficient mice showed more susceptibility to carcinogen-induced skin cancer as compared to the wild-type counterpart (63). Taken together, these studies indicate a tumor-suppressive role of ZBTB4 in cancer. However, more studies are needed as enough literature is not available on this protein.

#### ZBTB38

ZBTB38 can also bind to a single methylated CpG. It is also a bimodal protein that can bind to both methylated and unmethylated DNA. The *ZBTB38* gene that encodes the protein is located on chromosome 3. ZBTB38 plays roles in various cellular processes that include cell proliferation (186) and differentiation (187), control of DNA replication and genomic stability (188) as well as regulation of gene expression (173).

Results from a genome-wide association study (GWAS) done on prostate cancer patients revealed that polymorphisms in *ZBTB38* gene increase the risk of developing prostate cancer in men (64). In bladder cancer cells, ZBTB38 increased cell migration, invasion, and metastasis via regulating genes belonging to the Wnt/β-catenin signaling pathway but decreased bladder cancer cell proliferation (65). Depletion of *ZBTB38* increases the cytotoxic ability of DNMT inhibitors in different solid and hematological cancer cell lines by increasing the expression of Cyclin-Dependent Kinase Inhibitor 1C (*CDKN1C*) which opens up a new avenue to increase the therapeutic response to DNMT inhibitors (189).

#### ZFP57

This protein is encoded from the *ZFP57* gene located on chromosome 6. In addition to the Zinc finger domain, this protein also has an N-terminal Krueppel-associated box (KRAB) domain. ZFP57 preferentially binds to methylated CpGs within the TGCCGC hexanucleotide (190), and its primary functions include genome imprinting (191), regulation of gene expression, and cell signaling (192). Since it functions during early embryogenesis and is downregulated upon differentiation (193), it is also categorized as a stem cell transcription factor.

Ectopic expression of *ZFP57* enhanced the anchorage-independent growth properties of human fibrosarcoma HT1080 cells via the regulation of insulin-like growth factor 2 (*IGF2*) expression (194). In addition, knockdown of *ZFP57* gene caused suppression of HT1080 tumor formation while overexpression of the gene enhanced tumor formation in immunocompromised mice (194). The *ZFP57* gene expression has been shown to be elevated in patients with high-grade glioblastoma (66). Moreover, a recent GWAS revealed that *ZFP57* is a potential disease susceptibility gene for lung cancer development (67). Previous studies have shown that elevated IGF2 level is associated with a reduced survival rate in lung cancer (195). Since ZFP57 regulates *IGF2* expression, this axis may well be a potential target for lung cancer treatment.

#### KLF4

The Krüppel-like factor 4 (KLF4) is a 55 kD protein that has been demonstrated to function as one of the four factors needed for the generation of iPS (induced pluripotent stem) cells (196). The other functions of the protein include regulation of cell cycle (197), cell proliferation (198), DNA damage response (199), genomic stability (200), and apoptosis (201). KLF4 can recognize methylated CpG as well as unmethylated CpG or TpG within a specific DNA sequence (202, 203). It has been demonstrated that KLF4 binding to certain methylated CpGs attracts the recruitment of chromatin remodeling complex and thereby cause transcriptional activation (73), which is a paradigm shift from the common notion that DNA methylation readers mainly function as transcriptional repressors.

Both tumor suppressive and oncogenic function of KLF4 has been reported (69, 71, 72). KLF4 expression is downregulated in several malignancies like gastric cancer (68), colorectal cancer (69), and bladder cancer (70) where it mainly functions as a tumor suppressor gene. It has been shown that transduction of the *KLF4* gene using adenoviral vectors decreased proliferation and induced apoptosis of bladder cancer cells (70). As mentioned above, KLF4 also functions as an oncogene in some cancers. For example, both mRNA and protein levels of KLF4 are elevated during breast cancer progression (71). Knockdown of *KLF4* gene suppressed breast cancer cell migration, invasion, and colony formation *in vitro* and inhibited tumorigenesis in immunocompromised mice (72). KLF4 promotes cell adhesion and migration in glioblastoma cells (73). In addition, increased KLF4 has shown association with the progression and metastasis of human skin squamous cell carcinoma (74). In oral squamous cell carcinoma, *KLF4* showed dual functionality as a tumor suppressor as well as an oncogene (204). Taken together, these observations indicate a context-dependent role of KLF4 in cancer.

#### EGR1

The Early growth response protein 1, EGR1 (also known as ZIF268 or NGFI-A or KROX24) is a transcription factor that is induced following exposure to different cellular and external stimuli or stress signals (205, 206). The *EGR1* gene, located on chromosome 5, belongs to the category of immediate early gene and the encoded protein from this locus function in a plethora of cellular processes that include maintenance of synaptic plasticity (207), wound healing (208), inflammation (209), and differentiation (210). It recognizes and binds to the GCC(T/G)GGGCG consensus sequence near the target gene promoter regardless of the CpG methylation status (211, 212). In addition, the EGR1 binding affinity for cytosine (C) or 5 mC is much higher than that of 5-hydroxymethylcytosine (5 hmC) or 5-formylcytosine (5fC) (212). This implies that EGR1 can differentiate between oxidized and unoxidized C but not between methylated and unmethylated C moiety.

EGR1 expression is altered in several cancers. It functions both as a tumor suppressor and an oncogene depending on the type of cancer. The tumor-promoting effect of EGR1 has been demonstrated in Wilms' tumor (77) and prostate cancer (75). In contrast, it acts as a tumor suppressor in glioblastoma (80), fibrosarcoma (81), breast (78, 79), and lung cancer (82). Moreover, both male and female homozygous knockout (*Egr1*^−/−^) mouse cannot reproduce (213, 214). So careful considerations should be taken before pharmacologically targeting EGR1.

#### WT1

This protein is encoded by the Wilms' tumor 1 (*WT1*) gene located on chromosome 11. It was initially identified as a tumor suppressor gene that was inhibited in the most common form of pediatric kidney cancer called the Wilms' tumor 1 (83). Hence, it was named after that specific tumor even though later studies have demonstrated its presence in several other tissues and malignancies. It plays roles in the regulation of cell growth (215), differentiation (216), cell cycle (217), cell division and maintenance of genome stability (218). Like EGR1, WT1 also recognizes the GCC(T/G)GGGCG consensus sequence near the promoter of target genes (212). However, EGR1 and WT1 have differential binding sensitivity to the oxidative derivatives of 5mC (212).

WT1 may function either as a tumor suppressor or an oncogene depending on the type of cancer. Higher expression of WT1 has shown association with the poor prognosis of breast cancer (85), ovarian cancer (88, 89), leukemia (86), and head and neck cancer (87). In contrast, knockdown of *WT1* gene increased apoptosis of different cancer cell lines (219, 220). Moreover, WT1 has been ranked at the top position in the National Cancer Institute's (NCI) list of cancer antigens with the highest prioritization for vaccine development (221). The use of peptide vaccines against the WT1 antigen showed beneficial therapeutic outcomes in several clinical trials (222, 223).

#### CTCF

The CCCTC-binding factor (CTCF) is a well-known transcription factor that contains 11 highly conserved Zinc finger domains which allow it to bind at different locations on the genome (224) (Figure 4). One of the main functions of the CTCF protein is to act as a barrier/insulator to inhibit the interaction between the promoter and enhancer region (225). Other functions of CTCF include cellular context-dependent regulation of chromatin architecture, RNA splicing, and gene expression (225, 226). The DNA binding consensus sequence of CTCF contains CpG (227). It can bind to methylated DNA even though the binding preference for unmethylated DNA is much higher (228). For example, CTCF provides epigenetic stability to the retinoblastoma (*RB*) gene by binding at a specific region of the promoter sequence and thereby protecting the site from undergoing epigenetic silencing (181). Interestingly, CpG methylation at this particular region of the *RB* gene promoter leads to the loss of CTCF binding and promotes the binding of another methylation reader Kaiso (181). In this context, CTCF can be defined as a reader protein that can read methylated CpG but not necessarily binds to it.

CTCF can act as both tumor suppressor and oncogene depending on the type of cancer (229, 230). In ovarian cancer, CTCF expression is upregulated and shows association with poor prognosis (231). Higher expression CTCF in breast cancer cells has been shown to provide a survival advantage by inhibiting apoptosis (232). Depletion of the *CTCF* gene decreased proliferation and induced apoptosis of breast cancer cells (229). However, given that it plays a crucial role in the maintenance of chromatin architecture, targeting the downstream effectors of CTCF may serve as a more feasible option than targeting the protein itself.

### SRA Domain-Containing Proteins

**Figure 5:**
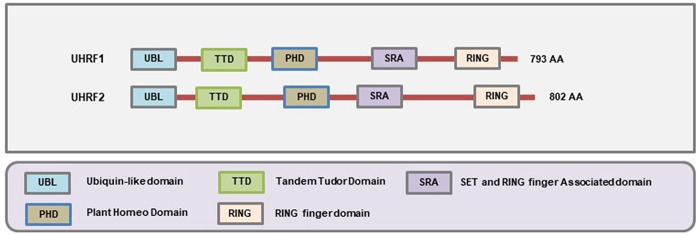
Schematic representation of the domains present in different SRA-domain containing proteins. The SRA domain allows them to bind to hemi-methylated DNA, the Ubl and RING domains are involved in ubiquitination, and the TTD and PHD domains allow to interact with the histones (Figure not drawn to the exact scale of the proteins).

The SRA family of methylation readers is comprised of two members: Ubiquitin-like with PHD and RING Finger domains 1 (UHRF1) and UHRF2. Both of these proteins contain at least five distinct functional domains that include the Ubiquitin-like Domain (UBL) (also known as the NIRF_N domain according to the NCBI Conserved Domain Database), Tandem Tudor Domain (TTD), Plant Homeodomain (PHD), SRA domain, and Really Interesting New Gene (RING) domain all of which confer tremendous functional complexities to these proteins (Figure 5). The N-terminal UBL domain and C-terminal RING domains are involved in the ubiquitin-proteasome system, the TTD and PHD domains can bind to the methylated histone proteins, and the SRA domain binds to the methylated CpG (233). The SRA domain is distinct from MBD/MeCP2 in their ability to recognize and localize to methylated sites on DNA since they possess a higher binding affinity toward the hemimethylated regions on the DNA (234, 235) while the MBD/MeCP2 domains tend to bind at the symmetrically methylated DNA (236).

#### UHRF1

The UHRF1 [also known as ICBP90 (human) or Np95 (mouse)] protein recognizes the hemimethylated DNA and subsequently recruits DNA methyltransferase 1 (DNMT1) to ensure that the sequence becomes faithfully methylated following replication (237, 238). It is a 793 amino acid containing protein encoded by the *UHRF1* gene located on chromosome 19 of the human genome. It was initially identified during the screening of proteins that can bind to the inverted CCAAT box (ICB2) region of topoisomerase Iiα promoter (239). During the earlier studies, the human version of the protein was called ICBP90, and the mouse homolog was called Np95. Hence, there was some confusion in the nomenclature of the protein (240). At present, it is most commonly referred to as UHRF1.

The ability of the SRA domain of the UHRF1 protein to bind the methylated DNA was first reported by Unoki *et al*. in human breast cancer cells where an elevated expression of the protein showed association with hypermethylation-mediated downregulation of early growth response 2 (*EGR2*) gene (241). More recent evidence showed that UHRF1 acts as a linker or adaptor between DNA methylation and histone modification (242). The interaction between UHFR1 and histone marks plays a crucial role in recruiting DNMT1 to the daughter stands. The other functions of the protein include regulation of cell cycle (243), cell proliferation (244), DNA damage repair (245, 246), apoptosis (as an anti-apoptotic protein) (247) as well as the progression of tumors (248, 249). Aberrant expression of the *UHRF1* gene has been found in different types of cancer like breast cancer (241), gastric cancer (95), hepatocellular carcinoma (250), renal cell carcinoma (251), squamous cell carcinoma (252), and osteosarcoma (253). Moreover, elevated levels of *UHRF1* in the blood of breast and gastric cancer patients has been shown to provide diagnostic and prognostic value as an independent biomarker (254, 255). Since UHRF1 is overexpressed in almost all major types of cancer, its role as a universal biomarker for cancer has also been proposed (256).

At the molecular level, UHRF1 acts as a transcriptional repressor since it binds to and recruits HDAC1 to the methylated CpG sites near the promoters of tumor suppressor genes (257). For example, UHRF1 binding at the promoters of *CDKN2A, RASSF1* (93), *KiSS1* (258), and *PAX1* (259) genes causes transcriptional repression of these genes. Loss of function studies showed that *UHRF1* depletion decreased cancer cell proliferation and increased apoptotic cell death (260, 261). Depletion of *UHRF1* also enhanced radio sensitivity of highly aggressive, triple negative MDA-MB-231 human breast cancer cells (262). Moreover, inoculation of *UHRF1* depleted MKN45 gastric cancer cells into immunocompromised mice showed a marked reduction in tumor volume and weight compared to the control tumors where *UHRF1* gene was not depleted (95). Polyphenols have shown promising effects in downregulating the expression of UHRF1 and thereby reduce tumor cell proliferation (263–265).

The UHRF1 protein also has some beneficial functions in cancer. It has been shown that UHRF1 directly binds the promoter region of the *MDR1* gene (also known as *ABCB1*) and thereby represses its expression (266). The *MDR1* gene encodes for P-glycoprotein (P-gp) which provides resistance to the cancer cells against various cytotoxic agents. It has been suggested that the UHRF1 mediated transcriptional repression of *MDR1* has the potential to overcome multidrug resistance during the treatment of breast cancer (266). Therefore, caution should be maintained while targeting UHRF1 for cancer treatment.

#### UHRF2

The UHRF2 [also known as NIRF or Np97] was initially identified as a protein implicated during the regulation of cell cycle (267). It is an 802 amino acid containing protein encoded by *UHRF2* gene located on chromosome 9 of the human genome. Even though it has a high degree of sequence similarity with UHRF1 at the amino acid level, it cannot functionally substitute UHRF1 in the maintenance of DNA methylation (268). In addition, structural studies have revealed that the SRA domain of UHRF2 protein but not UHRF1 preferentially binds to 5-hydroxymethylated DNA (5 hmc) (269). Therefore, UHRF2 has the unique ability to read and interpret at least three types of epigenetic marks that includes 5 hmC, 5 mC, and H3K9 methylation. It has been shown that UHRF2 can also enhance the enzymatic activity of Ten-eleven translocation methylcytosine dioxygenase 1 (TET1) (270). The expression of UHRF2 is higher in proliferating cells where PEST-containing nuclear protein (PCNP) acts as its substrate for ubiquitination (267, 271). Elevated expression of UHRF2 has been found in intrahepatic cholangiocarcinoma (99), colon cancer (98), breast cancer (100). Loss of function of *UHRF2* decreased breast cancer cell proliferation while forced overexpression of *UHRF2* gene into non-tumorigenic MCF10A breast cells induced cell proliferation (100). On the contrary, several groups have also reported on the tumor suppressor role of UHRF2 (101, 272). So, at present, it seems that UHRF2 plays roles as both oncogene and tumor suppressor depending on the cancer cell type. More detailed studies are warranted to establish its role in cancer and develop appropriate therapeutic strategies to attain clinically favorable outcomes.

## EPI-Therapies Targeting The MBPs In Cancer

Epigenetic therapies (“Epi-therapies”) targeting the components of the epigenome are emerging as potential therapeutic modalities for many pathological conditions. In cancer, several “Epi-therapies” are already being approved by the Food and Drug Administration (FDA) for the treatment of several hematological cancers. These drugs are mainly the inhibitors of DNA methylation and histone deacetylation. In the case of DNA methylation inhibitors, the two approved drugs are Vidaza and Dacogen (Decitabine) (273). While these two inhibitors can reverse the hypermethylated state at the promoters of tumor suppressor genes, they also induce the activation of several prometastatic genes (274). Therefore, targeting the DNA methylation readers/MBPs may serve a suitable alternative for the next generations of precision Epi-therapies.

**Figure 6:**
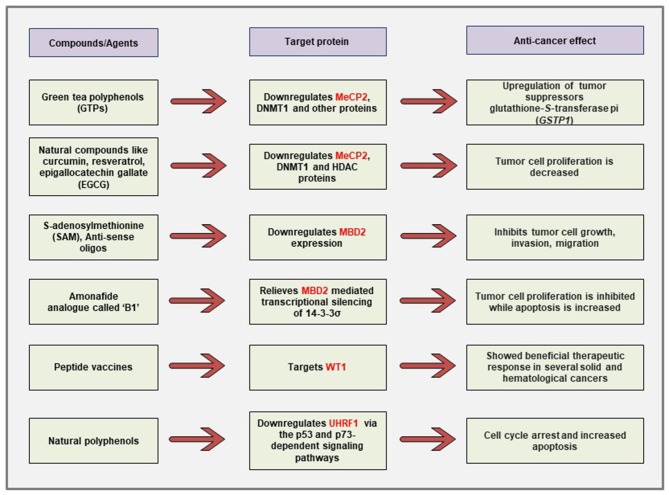
Schematic representation of the currently described anti-cancer strategies against different MBPs. Polyphenols obtained from natural compounds can downregulate the aberrantly expressed MeCP2, UHRF1 in cancer cells via differential regulation of cancer-related signaling pathways. The naturally occurring physiologic compound S-adenosylmethionine as well as anti-sense oligonucleotides can downregulate the elevated expression of *MBD2* gene and cause inhibition of tumor growth, invasion, and metastasis. Immunotherapy against WT1 antigen has shown promising effects in clinical trials for several malignancies.

Even though the field is still at its infancy, several studies have shown promising effects in terms of developing anti-cancer therapeutic strategies against the MBPs. A summary of the currently described anti-cancer strategies targeting different known MBPs is shown in Figure 6.

It has been demonstrated that treatment of prostate cancer cells with green tea polyphenols (GTPs) reversed the DNA hypermethylation-mediated silencing of the known tumor suppressor gene glutathione-*S*-transferase pi (*GSTP1*) through the downregulation of DNMT1, MeCP2, and several other MBD proteins (113). The authors have shown that GTP treatment causes demethylation at the promoter of *GSTP1*. Furthermore, chromatin immunoprecipitation (ChIP) assays revealed that GTP treatment also reduced the association of the transcriptional repressor MBD2 with Sp1 binding site that leads to the increased transcriptional activation of the *GSTP1* gene. In other studies, it has been shown that natural compounds like curcumin, resveratrol, guggulsterone, EGCG, withaferin A, and genistein can also cause the reversal of epigenetic state in cancer cells through the reduction of DNMT1, HDAC1, and MeCP2 protein expression (114).

Interestingly, polyphenols obtained from natural products have also been shown to decrease cancer cell proliferation through the downregulation of UHRF1 predominantly via the p53 and p73-dependent signaling pathways (263–265). *Limoniastrum guyonianum* aqueous gall extract (G extract), as well as luteolin, independently inhibited proliferation of cervical cancer HeLa cells by arresting the cells in G2/M phase and induced apoptosis through the inhibition of UHRF1 along with the upregulation of p16 tumor suppressor (275). In a mouse model of colon cancer, red wine polyphenols (RWPs) inhibited tumor growth, metastasis, angiogenesis, and increased apoptosis through the downregulation of UHRF1 and other proliferation markers like ki67, cyclin D1 (276). The UHRF1 expression has also been shown to be downregulated in mechanisms independent of the p53 and p73 signaling pathways. For example, in chronic lymphocytic leukemia patients, polyphenols from Bilberry extract (Antho 50) decreased UHRF1 expression and increased apoptosis via targeting the Bcl-2/Bad pathway (277).

The expression of *MBD2* gene was downregulated when the cancer cells were treated with the naturally occurring methyl group donor SAM that shows anti-proliferative and anti-metastatic effects (143, 144). This approach is particularly attractive because SAM is non-toxic to cancer cells and has been shown to cause downregulation of several other oncogenes and prometastatic genes without changing the expression of the known tumor suppressor genes *in vitro* and *in vivo* (278). Moreover, antisense oligonucleotides against *MBD2* gene decreased tumorigenesis in human lung and colorectal cancer cells both *in vitro* and *in vivo* (145). In human promyelocytic leukemia cells, an amonafide analog named B1 [chemical name: *N*-(2-(dimethylamino)ethyl)-2-aminothiazonaphthalimide] has been demonstrated to cause relief from the MBD2-mediated repression of 14-3-3σ tumor suppressor gene (279). Moreover, KCC07, a brain-permeable small molecule inhibitor of the MBD2 pathway, have been demonstrated to suppress medulloblastoma *in vivo* through the activation of BAI1/p53 axis (280).

Cancer immunotherapies have shown great promise as therapeutic strategies in patients. There are several forms of immunotherapies that include the use of checkpoint inhibitors, monoclonal antibody therapies, and vaccine immunotherapies against the tumor-associated antigens (TAAs) (281). With the advancements of cancer immunology, several TAAs have been identified, and one of them is a WT1 product (282, 283). Indeed, immunotherapies against the WT1 antigen showed promising outcomes in clinical trials on patients with several solid and hematological cancers (222, 223, 281, 284).

## Conclusion

This review summarized the roles of different MBPs based on the current literature with an aim to highlight their potential use as therapeutic targets and disease biomarkers. With the advances in the genome-wide binding studies, the specific functions of many MBPs are now clear even though more research is needed as some MBPs are still relatively less explored. In addition, the human proteome contains a wide array of Zinc finger motif-containing proteins, and recent evidence supports that many of these proteins may potentially bind to methylated DNA (5). However, in-depth experimental evidence is needed before classifying them as MBPs.

For the known family of MBPs, loss-of-function studies have aided to decipher their roles in different types of cancer. Since many of MBPs are cancer-type dependent, it opens a new avenue to develop targeted therapies against those subtypes. This will overcome the non-specificity issues related to currently approved epigenetic drugs like Decitabine and Vidaza, and thereby allow for a more specific approach to reduce cancer-associated morbidity and mortality. However, caution is warranted before the use of such targeted agents to avoid any potential negative ramification.

## Author Contributions

NM wrote the initial draft of the manuscript. SR revised it to its final version. Both authors have made a substantial intellectual contribution and approved it for publication.

### Conflict of Interest Statement

The authors declare that the research was conducted in the absence of any commercial or financial relationships that could be construed as a potential conflict of interest.
